# Static and Dynamic Recidivism Risk Factors of People Who Have Committed Child Sex Offenses in Sport

**DOI:** 10.3389/fspor.2021.624548

**Published:** 2021-07-05

**Authors:** Tine Vertommen, Helena Verhelle, Frederica M. Martijn, Minne De Boeck

**Affiliations:** ^1^Forensic Psychology, Thomas More University of Applied Sciences, Antwerp, Belgium; ^2^Social Epidemiology and Health Policy, University of Antwerp, Antwerp, Belgium; ^3^International Research Network on Violence and Integrity in Sport, Antwerp, Belgium; ^4^University Forensic Center, Antwerp University Hospital, Antwerp, Belgium; ^5^Collaborative Antwerp Psychiatric Research Institute, University of Antwerp, Antwerp, Belgium

**Keywords:** child sexual abuse, prevention, sexual violence, risk factors, extrafamilial abuse, recidivism

## Abstract

Current knowledge of people who commit child sex offenses (PCSO) in sport contexts is based on descriptive information from cross-sectional self-report studies of survivors and media coverage of court cases. In-depth scientific analyses of characteristics, interpersonal dynamics, and applied theories of sexual offending in sport are largely absent. This paper starts with a description of available Belgian data on PCSO in sport, coming from self-reports in community samples, reported cases in the media, and interviews with survivors. The main goal of this study is the analysis of treatment file information from 16 convicted PCSO in sport using two actuarial recidivism assessment instruments (STATIC-99R and STABLE-2007). Overall, the majority of the included PCSO's risk assessments indicated relatively low risk of sexual recidivism. Notable was the preponderance of high risk scores on items related to exclusively unrelated victims, male victims, sexual deviance, and the absence of an intimate relationship. Other static and dynamic factors related to the risk of sexual recidivism, e.g., (prior) non-sexual violent offenses, unknown victims, hostility toward women, lack of concern for others, and poor cooperation with supervisors were rated relatively low in this sample of PCSO in sport. The findings point toward the complex and nuanced patterns that underlie child sexual abuse in sport. The current findings bring us one step closer to filling in the puzzle of child sexual abuse in sport and will help inform evidence- and experience-based prevention and intervention efforts.

## Introduction

### The Global Problem of Child Sexual Abuse

Child sexual abuse (CSA) is a global problem of considerable extent. A meta-analysis on the worldwide prevalence of CSA, based on 331 independent samples reported in 217 publications between 1980 and 2008, revealed an overall self-report prevalence rate of 14% (Stoltenborgh et al., 2011). Self-reported CSA was more common among female participants (18%) than among male participants (8%). A systematic review focused on more recent studies, including 55 studies between 2002 and 2009, revealed prevalence estimates ranging from 8 to 31% in girls and 3 to 17% in boys (Barth et al., 2013).

Estimates of CSA prevalence vary greatly depending on the definitions used. Depending on country, discipline, or institution, definitions of sexual abuse can include narrow perspectives (e.g., only including genital penetration) vs. broad perspectives, including various contact sexual behaviors, such as fondling or oral penetration, as well as non-contact sexual behaviors, such as verbal sexual harassment or sextortion. In addition to the use of various definitions and differences of CSA worldwide, the legal age of consent to engage in sexual activity also yields differences in prevalence numbers of CSA.

The World Health Organization (WHO) defines CSA as

“the involvement of a child in sexual activities that he or she does not fully comprehend, is unable to give informed consent to, or for which the child is not developmentally prepared and cannot give consent, or that violate the laws or social taboos of a society. Child sexual abuse is evidenced by this activity between a child and an adult or another child who by age or development is in a relationship of responsibility, trust or power, the activity being intended to gratify or satisfy the needs of the other person” (World Health Organization, 1999, p. 15).

This paper uses CSA, and concurrently sexual offending against children, as a broad umbrella term for sexually transgressive behaviors, while recognizing that the varying use of definitions across research precludes broad generalizations.

Many victims of CSA do not disclose their experiences of abuse to anyone. If they do, it often takes a long time from the start of the abuse to their disclosure, and many refrain from making official complaints or reports. These aspects are likely responsible for the estimated high “dark number” of reported CSA (Schönbucher et al., 2012; Federale politie, 2018). Official statistics notoriously underestimate the actual prevalence of CSA, given the barriers for reporting and disclosure.

Official statistics and scientific data on CSA are scarce in Belgium. The Belgian legislation considers minors younger than 16 unable to consent, thus rendering all sexual activities with these minors as illegal in Belgium (Belgisch Strafwetboek, 1867). Bal et al. (2003) explored self-reports of sexual abuse among Belgian children aged 11–19. The results indicated that 10% of all Belgian children had experienced sexual abuse at least once in their lifetime. A 2010 study of 2,014 Belgian adults asked about their experiences of CSA -in total, 9% of women and 3% of men said they had experiences of CSA8 (Pieters et al., 2010). A large-scale study of sexual health in Flanders in 2013, known as the *Sexpert* study, explored experiences of CSA in 1,731 adult respondents and found that 22% of women and 11% of men reported having experienced sexually transgressive behaviors before the age of 18, ranging from unwanted sexual touching, to being forced to watch pornographic content, and having experienced rape and/or attempted rape (Buysse et al., 2013).

### CSA in Sport

How often does it happen? CSA does not occur in limited or specific contexts, but occurs across a multitude of environments and places, including the sports context. Prevalence studies on sexual victimization in sport are scarce. A systematic literature review yielded seven eligible studies on experiences of CSA in sport (Bjørnseth and Szabo, 2018), with only one study examining the prevalence of CSA in sport (Vertommen et al., 2016). Herein, an estimated 14% of children in sport reported experiencing sexually transgressive behaviors, including non-contact sexual harassment (i.e., verbal or non-verbal), contact sexual harassment (i.e., physical), and sexual abuse.

Studies focusing on people who commit child sexual offenses (PCSO)^1^ in sport are even scarcer. Only a few studies, some quantitative and others qualitative in nature, report on characteristics of PCSO in the context of sport. Self-report victim studies on sexual abuse in sport found that the majority was victimized by men (Vertommen et al., 2017). Interestingly, some studies find that peer athletes are most often identified as perpetrating the sexual violence, more often than coaches or other adult sport staff. Contrary to popular belief, studies across the world, mainly with student-athletes samples, found that the majority of sexual harassment in sport incidents are perpetrated by peer athletes: 88% in a UK study (Alexander et al., 2011), 86% in a Nigerian study (Elendu and Umeakuka, 2011), 33% in a Turkish study (Gündüz et al., 2007), and 23% in a Kenyan study (Rintaugu et al., 2014). In the same studies, the proportion of coaches who had perpetrated sexual harassment was remarkably lower: 8% in the UK study, 34% in the Nigerian study, 25% in the Turkish study, and 8% in the Kenyan study. The operationalization of sexually transgressive behaviors in these studies is different, which means a clear estimate of the prevalence of CSA cannot be determined.

Less is known about psychological characteristics of PCSO in sport. A qualitative study by Brackenridge and Fasting (Fasting and Brackenridge, 2009), based on interviews with 19 female athletes who were sexually harassed by their coaches, led to a preliminary typology development of coaches who sexually harass athletes: (1) the flirting-charming coach, (2) the seductive coach, and (3) the authoritarian coach. It should be noted that the sample size of this study was small, that all athletes in this study were at least 13 years old, but mostly older, and only included girls and women; thereby restricting the generalizability of this typology to other contexts. Another qualitative study examined court reports to determine the characteristics of PCSO in the context of sport to gain more knowledge about sexual violence, including sexual abuse, in sport (Fasting et al., 2013), but concluded that there was an absence of any “typical” perpetration profile.

### Available Data on PCSO in Sport in Belgium

To outline the current knowledge of the characteristics of PCSO in the Belgian sport context, several available data sources were consulted. First, information was drawn from the sole major prevalence study of sexual abuse in Belgian sport (Vertommen et al., 2017) that included both contact and non-contact types of (sexually) transgressive behaviors against child athletes below the age of 18. In a large general population sample of 2,044 Belgian (Flemish) adults who participated in organized sport before the age of 18, 341 (17%) reported experiences with sexually transgressive behaviors before the age of 18 (Vertommen et al., 2016). These participants were asked about the characteristics of the person who had perpetrated these behaviors. Results indicated that these sexual offenses were mainly perpetrated by men (91%), who were older than the victim (69%), and who had a position of authority within their respective sport organizations (Vertommen et al., 2017). While around two-thirds of the people reported to have committed these behaviors had coaching or managing positions, the other one third were (peer) athletes. The majority of the 75 persons reporting these experiences were women and were younger than 14 when the first instance of CSA happened (68 and 71%, respectively).

Second, information from newspaper articles on criminal cases was analyzed. In an unpublished Bachelor thesis, Bruyninckx (Bruyninckx, 2017) analyzed newspaper articles on 73 criminal court cases concerning sexual offenses in sport, of which 60 reported on sexual offenses committed against minors younger than 18. Of these, 63% of the cases reported on victims younger than 16. Within these cases, all prosecuted persons were men and were older than the victim, and 90% had a position of authority in the sport organization. More than one victim was identified in over half of the cases. About half of the cases concerned female victims only (49%) or male victims only (47%), and just 4% concerned both female and male victims.

Last, survivors' narratives provide qualitative information on the characteristics of CSA in sport. Following the European project “Voices for truth and Dignity: Combatting sexualized violence in sports,” nine Belgian survivors of CSA by their coach shared their narratives (Vertommen et al., 2019). All were extremely young when the abuse started, with an age range between 6 and 14, and an average age of 11. Most of them were girls (*n* = 7). In all narratives an older, male coach was reported as perpetrating the abuse, and, except in one case, all survivors reported also knowing about other children who were victimized by the same coach, before, during, or after their own victimization.

In summary, a high proportion of young and mostly female athletes experience sexually transgressive behaviors in sport. While we see a preponderance of this abuse perpetrated by older, male coaches, abuse perpetrated by peer athletes is not a rare occurrence.

### Explaining CSA

Persistent myths on PCSO still exist in most of society. While great advances have been made in the scientific domain of sexual offending and forensic psychology and psychiatry, taboo, stigmatization, and disinformation around sexual offending and recidivism remain big challenges. For example, of all types of delinquency, offenses breaching a person's sexual integrity, and in particular that of a child, are considered the most egregious (Quinn et al., 2004). Society often presumes that people who sexually offend are extremely dangerous and have a high risk of reoffending. Contrary to this belief, the base rate of sexual recidivism on a follow-up period of 5 years is in the 10–15% range (Hanson and Bussière, 1998; Harris and Hanson, 2004; Hanson and Morton-Bourgon, 2005), which is lower than commonly expected, and lower than most other types of offenses. In a recent study (Helmus L. M. et al., 2012), Helmus and colleagues found that, typically, sexual recidivism rates for PCSO are between 4 and 12% after 5 years, and between 6 and 22% after 10 years. Some longitudinal studies even demonstrate that it is more likely for PCSO to reoffend with a non-sexual offense than with a sexual offense (Hanson and Morton-Bourgon, 2004; Parkinson et al., 2004).

Most theories of sexual offending emphasize two psychological dispositions as main factors for the onset, persistence, and re-occurrence of sexual offending, namely antisociality and atypical sexual interests (Finkelhor, 1984; Hanson and Bussière, 1998), which can be further exacerbated by situational factors such as alcohol, strong emotions, and opportunity (Seto, 2018). Antisociality refers to propensity to engage in antisocial behavior, for instance typified by impulsivity, a willingness to cross social and legal boundaries, and risk taking (Seto, 2017). Atypical sexual interests can refer to non-normative sexual interests, preferences, or behavior. For instance, pedophilia (the persistent and recurrent sexual interest in prepubescent children), hebephilia (the persistent and recurrent attraction to pubescent children) (American Psychiatric Association, 2013), sexual regulation issues, or sexual coping (Seto, 2017). Contrary to common belief, pedophilia or hebephilia are not synonymous to sexual offending against children (Parkinson et al., 2004). According to various studies, 60 to 80% of the sexual offending against children is *not* committed by people with a pedophilic of hebephilic preference (Seto, 2008; Op goede grond, 2014). Many sexual offenses against children rather seem to be part of a generalist antisocial or criminal pattern, i.e., many people who commit a sexual offense have previously committed non-sexual offenses (Seto, 2017).

Next to psychological dispositions to commit sexual offenses against children, state facilitation factors and situational factors may play an important role. State facilitation factors are more dynamic and can differ from time to time within an individual, e.g., intoxication by alcohol or drugs, or strong emotions. Situational factors are related to opportunities to offend (e.g., being alone with a potential victim) (Seto, 2017).

Contrary the continuing myth that CSA is perpetrated by strangers in a cruel, aggressive way, much CSA occurs after a considerable period of friendly interaction that escalates to sexual abuse, also known as grooming (Mcalinden, 2006). More importantly, most CSA occurs within the home or by people who are known to the victim and with whom the victim has a close relationship (Kinderrechtencommissariaat, 2011; Tabachnick, 2013). The strong societal reaction to CSA and the stereotyping of PCSO as “dirty old men” or “monsters” reinforces both taboos and myths of sexual offending as well as contributes to keeping the barriers to report CSA high (Sanghara and Wilson, 2006). Prediction of sexual recidivism in child sex offending using risk assessment tools.

Recidivism is defined as the reversion to criminal behavior by an individual who was previously convicted of a criminal offense (Maltz, 2001). Because of the devastating impact on victims, their environment, and society at large, prediction of recidivism risk in PCSO is an important scientific, societal, and political issue. In this next section we will discuss sexual recidivism, which refers to any (sex) offense that is based on a sexual motivation, including contact and non-contact offenses (Marques et al., 1994; Harris and Hanson, 2004).

It is commonly thought that PCSO are at lifelong high risk for reoffending and remain dangerous. However, this assumed risk of reoffending in PCSO is overrated. Differences in recidivism rates vary considerably across settings and samples, and reoffending rates are affected by differences in follow-up time and sample selection (Helmus L. M. et al., 2012). Estimated recidivism risk also varies based on victim characteristics, e.g., recidivism risk is lower for people who have committed a sexual offense against a female, intrafamilial (incest) victim, than for people who have committed a sexual offense against a male, unrelated victim (Hanson, 2002). The risk of recidivism is the highest in the first year after release (Hanson, 2018). There is an emerging body of evidence that finds that sexual recidivism risk in people who have sexually offended decreases with age and offense-free time after release from custody, regardless of initial risk status (Barbaree et al., 2009).

The purpose of risk assessment is to determine the risk of recurrent (sexual) offending and to identify the specific factors that are of importance in determining treatment course to reduce recidivism risk (i.e., criminogenic needs) using validated risk assessment tools.

Risk assessments are generally based on both static and dynamic risk factors. Static risk factors are historical items (i.e., unchangeable items) relating to criminal history, age, and victim characteristics. These static risk factors are supplemented with dynamic risk factors (i.e., risk factors that are changeable over time), such as excessive sexual preoccupation, sexual deviance, emotional congruence with children, interpersonal problems, offense-supportive attitudes and beliefs, antisocial associates, resistance to rules, self-regulation difficulties, and substance use (Perkins et al., 1998).

Generally, three different risk assessment approaches are used in forensic psychology: unstructured clinical judgment, structured clinical judgment, and actuarial risk assessment. The first generation of risk assessments were unstructured clinical judgment. Clinicians individually assess the risk of recidivism based on their clinical (subjective) judgment, which might affect validity and reliability (Brown and Singh, 2014). Structured clinical judgment is an example of evidence-based assessment that emphasizes the use of research and theory, making use of a structured list of empirically validated risk factors, with room for other factors that the clinician deems relevant. The decision making is not a mathematical calculation, but a clinical estimation based on all items. The result of the assessment is not a numbered score but a judgment in terms “low,” “medium” or “high” risk of (sexual) recidivism (Goethals et al., 2020). Finally, actuarial risk tools are structured methods for combining static and dynamic risk factors into a total score or risk category. They represent highly structured risk assessment scales using empirically determined and thoroughly operationalized variables (Rettenberger and Craig, 2017). Actuarial risk assessment typically calculates the base rate, or the expected sexual recidivism rate, of the “typical” person who has committed a sexual offense with their relative risk score. This overall rate can then be adjusted up or down based on aggravating or protective factors (Helmus L. M. et al., 2012). Actuarial instruments provide empirically directed estimates of recidivism probabilities and are widely used in forensic treatment of PCSO (Hanson et al., 2013; Rettenberger and Craig, 2020).

Knowledge of and insight in recidivism rates are of paramount importance for treatment programs for PCSO since the primary goal of forensic treatment is desistance from committing new offenses. By estimating the risk of recidivism, the setting, intensity, and duration of treatment can be modified according to the risk level. Dynamic risk factors, which can be changeable over time, determine associated criminogenic needs, which in turn determine the focus of forensic treatment (van den Berg et al., 2020). It is important to note that most research into recidivism risk is based on new charges and/or convictism, i.e., recidivism records known to the justice, and it is unknown what amount of sexual recidivism goes undetected (Perkins et al., 1998; Harris and Hanson, 2004).

### Risk Factors for CSA in Sport

Analyzing risk factors for sexual recidivism might also be useful for the development and monitoring of prevention strategies at the level of sport organizations. Based on interviews with female athletes reporting sexual harassment in sport, Cense and Brackenridge proposed a temporal model of risk factors for sexual abuse in sport (Cense and Brackenridge, 2001), building on Cohen's Routine Activity Theory (Cohen and Felson, 1979). This theory emphasizes that crime occurs when three elements converge: a motivated offender, a suitable target, and the absence of a capable guardian. Cense and Brackenridge categorized risk factors for sexual abuse under risk factors having to do with coach, athlete, and the sport characteristics. Some of these risk factors seem to overlap with empirically validated risk factors for general sexual recidivism (e.g., lack of empathy, intimacy deficits, or disregard for other's boundaries), but others seem to be context specific and remain yet to be validated (e.g., dress requirements, amount of physical handling).

### The Aim of This Study

Current models and assessment tools in forensic psychology have not yet applied sexual offending research to the context of sport, and most previous research has focused on organizational and sociocultural factors in sports organizations. Up until today, very little is known about characteristics of PCSO in sport or risk factors related to the onset, persistence, or recidivism of sexual offending in this area. PCSO in sport, just like PCSO in other contexts, undergo criminal investigations, court trials, treatment, and supervision in general society, but may have idiosyncratic criminogenic or treatment needs, personality characteristics, or sexual deviancy issues that are different from the general PCSO population. This information can be very valuable in solving a piece of the puzzle of CSA in sport and informing future prevention and intervention efforts.

This study aims to combine insights from violence in sport research with insights from the practice of actuarial risk assessment for sexual recidivism and CSA prevention. This study analyzes the treatment files of 16 adult Belgian male PCSO in sport and examines static and dynamic risk factors related to child sexual reoffending in the context of sport. By using risk assessment tools, validated in general population samples of PCSO, we aim to identify which risk factors are important in sexual offending against children, specifically in the context of sport.

## Methods

### Data

This study is based on the casefiles of 16 PCSO in sport, recruited from two outpatient forensic treatment facilities in Belgium that treat adults who have committed or are at risk of committing sexually transgressive behaviors. Cases were selected based on the following inclusion criteria: being convicted for a hands-on sexual offense against at least one child who was aged 15 or younger at the start of the sexual offense and having at least one victim who they met in the context of organized sport. All participants were men with an average age of 30 at time of the first offense (range 12–44 years old) (see Table 1). At the most recent risk assessment, which took place at the end of treatment, or while still in treatment, the average age of the participant was 46 (range 26–61 years old). Most participants were single with no children. Ten different sports were represented in this sample. The majority of participants had a position of authority in the sport organization (*n* = 11). The average number of identified victims was 5 (range 1–13) and the average age of the youngest victim was 11 (range 2–14 years old). Ten participants offended against male victims, and seven offended against female victims, which means one participant offended against both male and female victims.

**Table 1:** Characteristics of PCSO in sport, offenses and victims.

**Pseudonym**	**Age at first offense**	**Age at last risk assessment**	**Civil status**	**Sport**	**Role in the sport organization**	**Number of victims**	**Age of youngest victim**	**Victim's sex**
Seppe	12	31	Single, no children	Sport diving	Member	5	6	Male
Stan	18	50	Single, no children	Basketball	Member	7	2	Male, Female
Jan	21	25	Single, no children	Basketball	Member	2	14	Male
Lars	21	26	Single, no children	Swimming	Coach	2	13	Female
Max	25	46	Single, no children	Martial Arts	Club owner	3	10	Male
Jef	26	42	Single, no children	Kayaking	Coach	4	5	Male
Kurt	28	61	Married, 2 children	Swimming	Instructor	13	11	Female
Lucas	31	56	Single, no children	Martial Arts	Coach	7	10	Male
Davy	32	40	Single, no children	Pétanque	Member	1	13	Male
Willy	34	61	Single, no children	Martial arts; football	Coach & club delegate	2	14	Male
Tom	35	44	Divorced, 2 children	Martial Arts	Coach	5	11	Female
Thomas	35	39	Single, no children	Diving	Member	1	12	Female
Jarne	36	52	Married, no children	Horse racing	Coach	7	14	Female
Maurice	39	50	Divorced, 1 child	Horse racing	Coach	5	14	Female
Ronald	43	58	Single, no children	Sailing	Coach	10	13	Male
Mark	44	48	Married, no children	Football	Coach	1	14	Male

### Materials

All participants had previously been convicted of a child sexual offense and concurrently followed or had completed a psychiatric and psychological treatment in a Belgian forensic outpatient treatment facility. The treatment was a condition for parole, probation, or alternative penalty. The case files included police reports, court case records, victim statements, treatment records, and medical and diagnostic assessments. This information was used to assess their risk of recidivism using two actuarial risk assessment tools that are well-validated and widely used in assessing sexual recidivism risk, and should be favored over other approaches in recidivism assessment (Hanson and Morton-Bourgon, 2009).

#### STATIC-99R

The STATIC-99R (Phenix et al., 2000) is an empirically derived actuarial risk assessment tool designed to predict sexual recidivism in men who have committed a sexual offense. The STATIC-99R was validated in 2014 in the Netherlands and is thus available in Dutch (Smid et al., 2014; Phenix et al., 2016). The STATIC-99R consists of 10 historic items assessing demographic information (age at release, relationship history), sexual criminal information (prior sexual offenses, any male victims, any unrelated victims, any stranger victims, any non-contact sexual offenses), and general criminal information (prior sentencing dates, index offense including non-sexual violence, prior non-sexual violent offenses). The absence of the risk factor is scored with a “0,” the presence of the risk factor is scored with a “1,” i.e., lower scores indicate lower risk of reoffending. Current age is scored between “−3” and “1,” with people 40 or older scoring “−1” and people 60 or older scoring “−3” (i.e., indicating that older age is related to a lower risk of reoffending). The risk score for the number of previous sexual offenses is based on both the number of charges as well as convictions and varies between “0” and “3.” The risk score for index sexual offense is based on the most recent sex offense for which the person was charged, arrested, or convicted. A meta-analysis found a moderate relationship between the STATIC-99R and sexual recidivism (AUC.69) (Helmus L. M. et al., 2012).

#### STABLE-2007

The STABLE-2007 is a risk assessment tool using 13 dynamic risk factors related to sexual self-regulation, general self-regulation, social relationships, intimacy deficits, and cooperation with supervision (Fernandez et al., 2012). Because of the potentially changeable nature of dynamic factors, the STABLE-2007 is often used to inform treatment targets and criminogenic needs (Hanson et al., 2017). The scoring manual is available in Dutch (van den Berg et al., 2007). Risk factors are scored with a “0” indicating “no concerns” or “not present,” a “1” is given when there is uncertainty about whether the factor is present or when the factor is present but not strong enough to justify a maximum score of 2, and a “2” suggests sufficient concerns within this risk domain (Fernandez et al., 2012). Higher scores on the STABLE-2007 indicate a higher risk of reoffending. Validation studies show a moderate predictive value of the STABLE-2007 for sexual recidivism (AUC.67) (Rettenberger and Craig, 2017).

#### Static-Stable

The static and dynamic risk scores, determined by the score on the STATIC-99R and STABLE-2007, can be combined to get an overall assessment of the risk and needs level of a PCSO. Hanson and colleagues (Hanson et al., 2017) make use of the standardized risk framework, which is a method for quantifying risk according to standardized risk levels for sexual offending (Hogan and Sribney, 2019). The risk levels are: Risk level I, *very low risk*, which is similar to people with non-sexual criminal histories; Risk level II, *below average risk*, which is higher than the very low (I) risk profile but lower than the average (III) risk profile; Risk level III, *average risk*; Risk level IVa, *above average risk*, which is approximately two times the average risk (III); and Risk level IVb, *well above average risk*, which is approximately three to four times higher than the average risk (III) (Brankley et al., 2017).

### Procedures

In order to gather sufficient data for this study, two outpatient treatment facilities in Belgium that treat adults who have committed or are at risk of committing sexually transgressive behaviors were contacted. Based on the inclusion criteria of this study (conviction for contact child sexual offenses in the context of sport, victim's aged 15 or younger), a total of 16 treatment files were selected. Treatment files were anonymized by the treatment facilities and the researchers signed a confidentiality agreement before accessing the files. The files were not digitalized and only accessible in the treatment facilities. Files were consulted in the treatment facilities between February 2017 and February 2018. Notes, required to substantiate the scores, were preserved. Depending on the treatment facility protocols, risk assessment tools are regularly scored, or are scored depending on key dates in their trajectory (e.g., at the end of the parole dates). Scores in this study are based on end-of-treatment scores (*n* = 8) or scores during ongoing treatment (*n* = 8). Assessment scores were checked with the clinician responsible for the respective file and discussed within the research team, which included two persons professionally trained in scoring the STATIC-99R and STABLE-2007. Assessment was performed following the guidelines provided in the Dutch manuals of the tools (van den Berg et al., 2007; Smid et al., 2014). Raw scores on both instruments were combined, following the guidelines for the five level risk communication (Hogan and Sribney, 2019). These instruments are validated on their intended target group (i.e., men convicted of contact sexual offenses). By no means are these instruments intended to be used for screening in a general population.

Short vignettes describing the offense characteristics were composed, consisting of information on the persons' pseudonyms, type of sport, age, gender, number of victims, and some characteristics of the offense circumstances. The study protocol was submitted to and cleared by the Ethics Commission of the University of Antwerp (code 16/50/550).

## Results

The following sections give a descriptive analysis at group level per risk factor item for each of the risk assessment instruments.

### STATIC-99R

#### Demographic Items

The age of release from index sex offense *(STATIC item 1)* in this sample of PCSO in sport is relatively high, mostly between 40 and 60 years old, which indicates a decreased risk of recidivism (see Table 2). Regarding their civil status *(STATIC item 2)*, 10 out of the 16 PCSO in sport had never lived with an adult intimate (sexual) partner for 2 years or longer, prior to release from the index offense.

**Table 2:** Risk assessment scores per item (*N* = 16) on the STATIC 99-R.

**Item**	**Risk factor**	**−3**	**−1**	**0**	**1**	**2**	**3**
1	Age at release from index sex offense	2	12	–	2		
2	Ever lived with an intimate partner – 2 years			6	10		
3	Index non-sexual violence – Any convictions			14	2		
4	Prior non-sexual violence – Any convictions			16	–		
5	Prior sex offenses			11	4	–	1
6	Prior sentencing dates			16	–		
7	Any convictions for non-contact sex offenses			12	4		
8	Any unrelated victims			–	16		
9	Any stranger victims			16	–		
10	Any male victims			6	10		

*- indicates none of the participants were assessed to score at that particular level. Blank spaces indicate items are not measured on those scoring levels*.

#### Criminal History

Two PCSO in sport had any prior or index offense convictions of non-sexual violent offenses *(STATIC item 3 and 4)*, and five PCSO had previous charges or convictions for sexual offenses (*STATIC item 5*). Of these, four PCSO had one or two charges or one conviction for a sexual offense prior to the index offense, and one PCSO had eleven previous sexual offense charges, but no convictions. None of the PCSO in this sample scored at elevated risk for having multiple prior sentencing dates (*STATIC item 6*), meaning all of them had three or less previous sentencing dates. The *STATIC item 7* considers any (also other than the index offense) non-contact sexual offenses and mainly refers to the possession or distribution of CSA materials (commonly, but misleadingly, referred to as “child pornography”). Four PCSO in this sample had previous convictions for non-contact sex offenses.

#### Victim Characteristics

The last three items in the STATIC-99R concern victim characteristics. Having unrelated, stranger, and male victims is related to increased risk of recidivism in PCSO. The scoring of these items is based on all available, credible information, including self-report, victim statements, and collateral information. The average number of victims in this sample was five. This does not include victims of non-sexual violence, possession of CSA materials, or visiting sex workers. As expected, all PCSO in sport in this sample had at least one unrelated victim, i.e., the child in the sport context *(STATIC item 8)*. Two PCSO had both unrelated and related (i.e., familial) victims. None of the participants had any unknown (i.e., stranger) victims *(STATIC item 9)*. The majority of participants (*n* = 10) had at least one male victim *(STATIC item 10)*.

### STABLE-2007

#### Social Contacts

The first item in the STABLE-2007 captures positive and negative influential social contacts (see Table 3). None of the PCSO had negative social contacts at time of assessment. Four PCSO had insufficient positive social contacts.

**Table 3:** Risk assessment scores per item (*N* = 16) on the STABLE-2007.

**Item**	**Stable - 2007**	**0**	**1**	**2**	**DNA**
1	Significant social influences	12	4	–	
2	Capacity for relationship stability	5	2	9	
3	Emotional congruence with children	6	5	–	5^*^
4	Hostility toward women	16	–	–	
5	General social rejection/loneliness	6	9	1	
6	Lack of concern for others	13	3	–	
7	Impulsive acts	13	2	1	
8	Poor problem-solving skills	9	4	3	
9	Negative emotionality/hostility	12	4	–	
10	Sex drive/pre-occupation	12	2	2	
11	Sex as coping	16	–	–	
12	Deviant sexual interests	1	6	9	
13	Co-operation with supervision	13	3	–	

* *This item can only be scored when victims are below the age of 14. Five PCSO in sport have victims older than 14*.

*- indicates none of the participants were assessed to score at that particular level. Blank spaces indicate items are not measured on those scoring levels*.

#### Intimacy Deficits

Eleven PCSO's showed increased risk concerning their incapacity of having and maintaining an intimate sexual relationship with an adult partner (*STABLE item 2*). This finding mirrors the scores on the STATIC item 2 *Ever having lived with an intimate partner for more than 2 years* but additionally evaluates the quality of the PCSO's current relationship. The presence of a safe and healthy adult relationship is considered as a protective factor against sexual recidivism.

Some PCSO show emotional congruence with children, which refers to a heightened emotional identification with children and the feeling of being emotionally connected to children. PCSO with high emotional congruence with children may find themselves to connect more easily with and feel that they understand and are understood better by children than by adults or may be childlike themselves in terms of interests and leisure activities. Five PCSO showed moderate emotional congruence with children (*STABLE item 3*), and six did not. As this item is only scored when there are identified victims who are 13 or younger, this item was not scored in the remaining five cases.

The sample scored in the direction of low risk regarding items representing lack of empathy or general antisocial attitudes. None of the PCSO in this sample showed hostility toward women *(STABLE item 4*). The majority of the PCSO in this sample (*n* = 13) showed adequate concern for others *(STABLE item 6)* with only three PCSO showing moderate problems in terms of lack of concern for others. However, 10 out of 16 PCSO indicated experiencing feelings of general social rejection and loneliness (*STABLE item 5)* and were feeling insecure in their connection to their social environment at the time of the assessment.

#### General Self-Regulation

PCSO in this sample reported varying levels of risk related to cognitive problem-solving skills (*STABLE item 8*), with four PCSO showing moderate issues and three PCSO showing severe deficits related to problem solving skills. Contrarily, risk related to impulsivity (*STABLE item 7*) was absent in most of this sample, with only two PCSO showing moderate and one PCSO showing severe problems related to impulsivity. Negative emotionality/hostility, or the tendency to feel victimized and generally mistreated by others, was rated relatively low in this sample (*STABLE item 9*), and only 4 PCSO showed moderate signs of negative emotionality.

#### Sexual Self-Regulation

The item “Sexual deviant interests” (*STABLE item* 12) can be retrospectively scored based on victim characteristics. Scores can be determined by either the total number of victims, the total number of deviant victims (i.e., in this sample, boys younger than 14 or girls younger than 13), by results of specialized testing, and/or by self-reported deviant sexual interest. Having two to seven victims results in a score “1” and having eight or more victims results in a score “2.” Having one deviant victim results in a score “1” and having two or more deviant victims results in a score “2.” The majority of PCSO in sport in this sample had elevated scores on this item, with nine PCSO scoring at highest risk, and six PCSO scoring at moderate risk. Further, two PCSO experienced severe problems and two PCSO experienced moderate problems related to sexual preoccupation *(STABLE item 10)*, such as excessive and/or high sex drive, frequent use of sex websites, porn collections, impersonal sexual behavior, or fetishistic sexual behavior negatively impacting their relationships or other domains in their life. Based on the case file information, the other PCSO did not report problems related to sexual preoccupation at the moment of the assessment, and none of PCSO in this sample used sex as coping at the time of assessment (*STABLE item 11*).

#### Cooperation With Supervision

Most participants cooperated well with supervision, such as probation officers or therapists *(STABLE item 13)*, indicating no issues in terms of adherence to parole conditions or treatment programs.

### Risk Categories and Combined Risk

**Figure 1:**
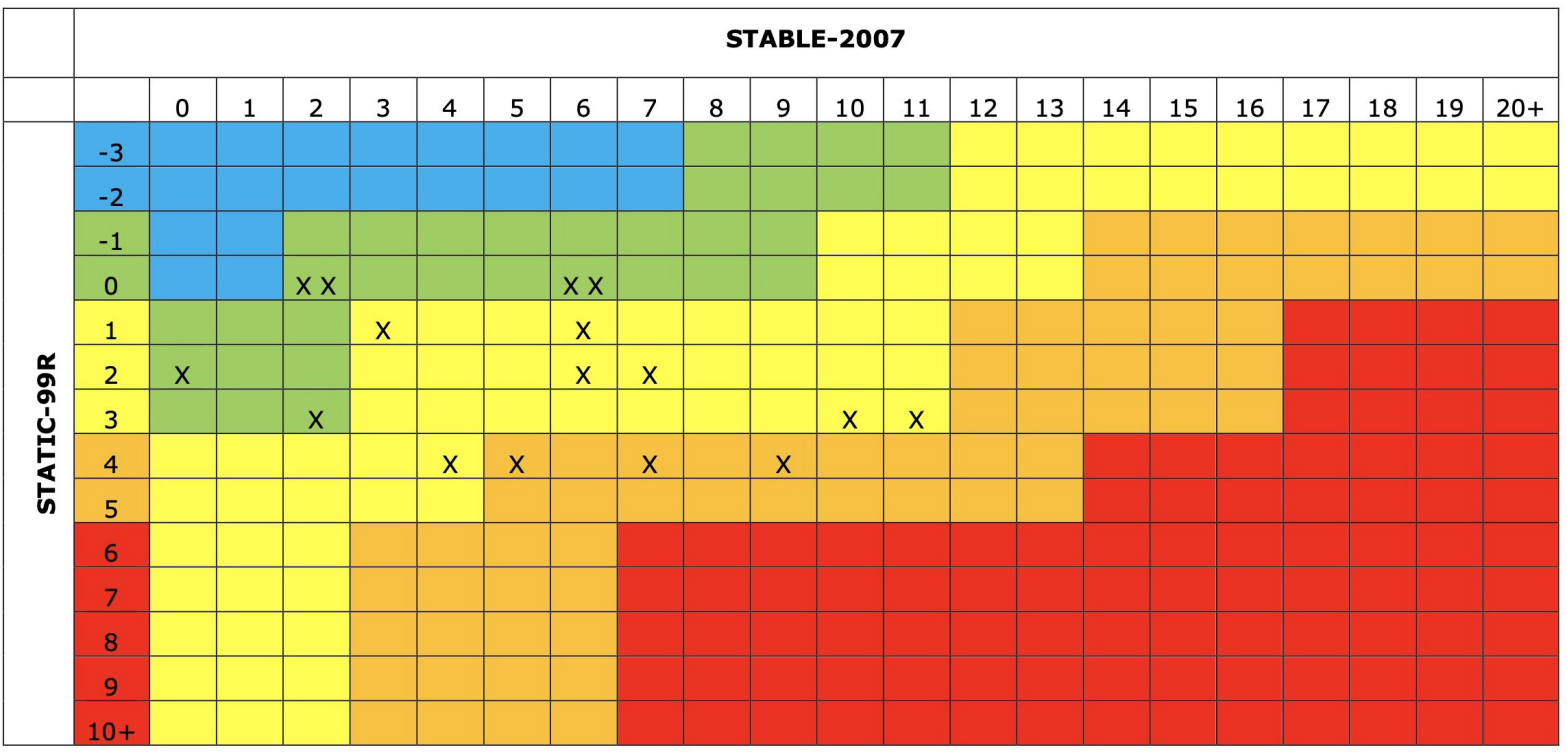
Combined risk categories. Blue: Risk level I, very low risk, which is similar to people with non-sexual criminal histories; Green: Risk level II, below average risk, which is higher than very low (I) risk profile but lower than average (III); Yellow: Risk level III, average risk level; Orange: Risk level IVa, above average risk, which is approximately two times the average risk (III); and Red: Risk level IVb, well above average risk, which is approximately three to four times the average risk (III) (Brankley et al., 2017).

The total scores of the STATIC-99R and the STABLE-2007 are combined to calculate the overall risk of sexual recidivism based on the static and dynamic risk factors. The majority of PCSO in this sample were assessed at a Below Average *(n* = 6) or Average Risk (*n* = 7) of sexual recidivism (see Figure 1). The Below Average Level (Level II) means that on average 5.3% of PCSO assessed as this risk level reoffend with a sexual offense within the next 5 years. An Average Risk (Level III) means that on average 7.5% of PCSO assessed at this risk level reoffend with a sexual offense. Three PCSO showed an Above Average Risk (Level IVa) of sexual recidivism, related to a 13.6% sexual recidivism risk in a follow-up time of 5 years (Brankley et al., 2017).

## Discussion

This study assessed case files of 16 PCSO in sport to assess static and dynamic risk factors related to sexual recidivism. The assessments indicated a *below average* or *average* recidivism risk for the majority of the PCSO in this sample, which means that 5.3–7.5% of PCSO assessed at this risk level reoffend with a sexual offense within 5 years after release from custody. Only three PCSO were assessed at an *above average* risk level, corresponding to a sexual recidivism risk of 13.6% within 5 years (Brankley et al., 2017).

Some interesting patterns emerged from the analyses of the individual static and dynamic risk factors in this sample of PCSO in sport. On the one hand, PCSO in this sample seem to have low risk scores related to markers of antisociality (such as sexual or non-sexual criminal offense histories, little use of violence in during their index sexual offenses, low impulsivity, and low hostility), showed limited emotional congruence with children, and reported few problems related to sexual preoccupation or using sexual coping strategies at the time of the assessment. On the other hand, the majority of this sample of PCSO in sport showed higher risk scores related to markers of deviant sexual interests (such as offending against boys, unrelated, and multiple victims), had significant problems related to intimate adult relationships, and experienced feelings of social rejection, which are all risk factors for sexual reoffending.

Interestingly, most PCSO were relatively old (at least 40 or older) at their time of release. Older age is related to lower chances of sexual recidivism (Helmus L. et al., 2012). The relatively old age at time of release within this sample may have different explanations, such as a late start of offending behavior, a long time between the start of the sexual offending behavior and the victims' disclosures (and thus the start of the judicial process), the clustering of several sexual offenses against different victims over the span of several years in the index offense, and/or a long judicial and treatment process (Rettenberger and Craig, 2017).

While the PCSO in this sample had no or few known previous sexual or violent offenses, it should be noted that these only include officially registered (charges or convictions of) previous offenses. It is possible that previous offenses were not reported to the police, and thus not officially registered by the justice system. Alternatively, some sport organizations prefer to informally deal with reported transgressive behaviors within their organizations, which contributes to the hypothesized high dark number of sexual offenses. Furthermore, CSA, especially when the person who commits the sexual abuse is known to the victim, is seldomly a sudden or violent occurrence. The grooming process, that is commonly described as a precursor to CSA in sport (Brackenridge, 2001; Owton and Sparkes, 2015), often includes preparatory behaviors leading up to the offense that are hardly detectable due to the intimate and secret relationship of trust that is built between the adult and child. Sport often includes physically intimate behaviors, during practice, training or circumstantial activities such as changing and showering, but these may also provide a guise for transgressive behaviors and escalation thereof. The further widespread normalization of physically and sexually transgressive behaviors in sport (Parent and Fortier, 2018) may also contribute to transgressive behaviors going unrecognized by victims, guardians, or organizations, or a further minimization or concealment by sport organizations of inappropriate behaviors that lead up to the offense.

Poor or inadequate problem-solving skills and impulsivity may be related to sexual reoffending. Poor cognitive problem-solving skills might increase the risk of recidivism as this impacts the capacity for problem and solution evaluation. Impulsivity may increase recidivism risk if someone does not oversee the long-term consequences of their behavior, or if someone does not have adequate skills to inhibit impulsive wants or needs. In order to be assessed at elevated risk scores on these items on the STABLE-20007, problems in these areas should be present across a number of settings apart from the sexual offense. While poor problem-solving skills and impulsivity are often related to each other, this sample surprisingly reported almost no problems related to impulsivity and moderate problems related to problem solving skills. CSA behaviors can be, but are not necessarily, impulsive acts (Brackenridge and Fasting, 2005; Mcalinden, 2006). The findings in this study might suggest that the PCSO in sport of this sample did not commit their sexual offenses because of problems with impulse control, which would be in line with previous research that indicates that most sex offending in sport by people in positions of power is preceded by a grooming process (Brackenridge and Fasting, 2005; Mcalinden, 2006; Owton and Sparkes, 2015).

The findings that this sample of PCSO included more participants with one or more male victims than participants with only female victims was surprising as findings of the Belgian prevalence study and court files analysis indicated that the victimization rates of girls and women is higher than of boys and men (Vertommen et al., 2016; Bruyninckx, 2017). It is possible that the dark number of sexual victimization among boys and men in sport is even higher than those of girls and women, given the greater taboo of disclosure of male-on-male sexual offending, or, alternatively, the normalization of sexualized behavior between men (Hartill, 2009). It is not possible to determine whether the high number of PCSO with male victims in this sample is a reflection of the sexual gender attraction of the PCSO, or due to situational factors. For example, situational factors might be that boys participate at higher rates in sport compared to girls (Sport Vlaanderen, 2020), or there are more situational opportunities for sexual offending to occur between male coaches and male athletes, for instance as changing and shower rooms are shared. It is also possible that sexual victimization of girls is problematized more than sexual victimization of boys in our society. The downplaying and minimizing of male sexual victimization may conserve the taboo around reporting and may lead to less awareness and vigilance toward boys as possible victims of abuse.

The number of victims identified in the cases of these 16 PCSO is high, with five victims on average. While the number of victims is not an unambiguous predictor of recidivism, it might give us useful information related to the predation of sexual offending. The high number of victims per PCSO combined with the relative absence of previous convictions might plausibly indicate there were multiple opportunities across longer periods of time for PSCO in sport to offend against several victims, while their offenses were undetected.

Stigmatization and social rejection of PCSO in sport after they are arrested, charged, or convicted of a sexual offense is not uncommon, but nonetheless concerning for the mental health of PCSO. PCSO suffer stigmatization and rejection of their sport's communities (Cortoni et al., 2017). The vignettes of the 16 PCSO in this sample revealed that many of these people had a fulfilling social lives that were primarily centered in and around the sport club predating the offense. When CSA cases are disclosed or reported to official authorities, leading to criminal investigations and possible convictions, this has a major impact on the individual and social life of the person who is accused of CSA. Not surprisingly, the risk assessment also indicated problems in the social areas of the PCSO in this sample, even though results seemed somewhat contradictory: when assessing the social contacts of PCSO, it was found that most of the PCSO in this sample still have sufficient positive and/or neutral social influences, indicating they are sufficiently embedded in a social structure. However, their perceived social isolation or rejection scores indicates that most of them do not feel adequately embedded and experience some form of insufficiency of their social life. This might reflect the possible loss of a widespread social network via the sport organization. It should be noted that such dynamic risk factors can change over time and could have been different at the time of the offense.

It is important to note that some of the established general risk factors for sexual reoffending are not present in this sample. Hostility toward women was not notably present in this sample and is likely more closely related to sexual offenses against adult women than to sexual offenses against children. Also, there were few signs of sexual preoccupation and sexual coping, which indicate problems related to sexual regulation and being able to deal with stressful emotions in a non-sexual way. We should note, however, that these items were scored at the time of assessment and not retrospectively at the time of the offense. It is possible that these scores have changed over time, for instance through treatment interventions, and would have a higher (or lower) score if they were assessed at the time of offense.

### Recommendations for Prevention of CSA in Sport

The findings in this study show that PCSO in sport, just like PCSO in general, are not a homogenous group. It is also clear that this sample of PCSO in sport score lower on the antisocial factors and do not have extensive criminal histories. On the contrary, looking at their social embedding, the PCSO in this sample appear to be largely normally functioning, socially accepted people with often significant roles in the sport organization. While there are no rational reasons for not performing a criminal record check on all adults working with children in sport, we should be acutely aware of its limited effectiveness. A criminal record check does not provide protection against people who will commit their first offense, or people who have committed offenses that are not reported or noticed by the justice system (Abrams and Bartlett, 2019). The PCSO in sport of this sample had indeed often committed multiple sexual offenses against multiple victims that remained undetected for a long time.

Prevention efforts for CSA are generally divided into three types: primary, secondary, and tertiary prevention. While definitions differ, researchers such as Wortley and Smallbone (Wortley and Smallbone, 2006) define primary prevention and secondary prevention as having the purpose of preventing CSA before it occurs. Primary prevention focuses on the general population or sub-groups of the general population, and secondary prevention focuses on specific groups at risk. Tertiary prevention on the other hand is aimed at preventing the reoccurence CSA by focussing on individuals who have engaged in sexual offending against children (Smallbone et al., 2008; Knack et al., 2019). Primary and secondary prevention strategies are most useful when combatting sexual violence in sport, as we are often not even aware of CSA in sport, and as we want to prevent children in sport for being victimized in the first place. This is why prevention efforts cannot solely focus on tertiary efforts, Empowering athletes, coaches, and their entourage by educating them about adequate communication about sexual integrity, personal boundaries, and respect will have more far-reaching impact than investing in reactive measures only (e.g., criminal background checks). Involving all stakeholders in sport in primary prevention initiatives, such as proper awareness raising, educational activities about codes of conduct, and information on the availability of reporting mechanisms, will not only empower athletes, but also stimulate bystanders to appropriately intervene when necessary. The clear and safe availability of reporting mechanisms, safety procedures, and local points of contact, as part of the secondary prevention strategy, might lower the threshold to report or disclose sexual misconduct (Mathews et al., 2016). The world of sport shows to be a conducive climate for all sorts of integrity violations. Situational factors at the organizational level, e.g., changing and shower rooms, hotel rooms, car drives, or time spent alone between a coach and a young athlete all create ample practical opportunities for abuse to take place. Sport organizations should undergo continuous evaluations and subsequent behavioral adjustments to minimize these potentially risky situations (Wortley and Smallbone, 2006).

Available helplines for people who experience deviant sexual thoughts or worry about their own behaviors, and for people who are worried about other people's behavior, such as *Stop it Now!* (Horn et al., 2015) should be more thoroughly promoted in the sport context. By offering help to people who are worried about themselves, and by spotting signs of abuse early and starting a conversation, (potential) CSA can be prematurely detected and stopped. It is not up to the sport organization to reinvent the wheel, as general secondary and tertiary prevention services for people who are (potentially) victimized or who are perpetrating transgressive behaviors have adequate tools available. Investing in open communication about risks, responsibilities, and safety of children requires a shift in mentality and a willingness to acknowledge the everlasting need of child protection in sport.

Tertiary prevention focuses specifically on preventing PCSO from reoffending. Banning all PCSO from sport forever could theoretically prevent this population from reoffending in the sport context, but practically is not manageable or controllable. Also, denying all convicted PCSO to return to sport is not a waterproof method to prevent sexual offenses from happening, as those who reoffend only make up a small portion of the total of offenses that take place. The lifelong ban of PCSO from sport also means their social reintegration is significantly stunted, which may lead to a lack of meaningful professional or leisure activities, social rejection, loneliness, lack of positive social contacts, isolation, and stigmatization, which are all related to an increased risk of recidivism (de Vries Robbé et al., 2015). Recidivism models, such as the Good-Lives-Model, stress the importance of meaningful life goals and working toward positive and healthy ways to meet personal needs (Ward and Brown, 2004). Taking away protective factors, such as positive social contacts and meaningful activities, can lead to an accumulation or acceleration of factors related to reoffending risk. Formulating a strategy in which reintegration in sport is possible, but controlled (e.g., not in a coaching role, only working with adult athletes, always having someone else in the room, under strict conditions of social control mechanisms) provides more opportunities to balance the vulnerable equilibrium between both societal and individual interests of reintegration. However, fewer risks of reoffending and an increased embeddedness of people who have committed sexual offenses within society eventually decreases risks of reoffending and increases safety for everyone. Unfortunately, thorough public debate about safe reintegration of PCSO in sport is currently hindered by negative or false beliefs about sexual recidivism rates and other stigmatizing myths about this population. A better understanding of the risks and needs of this population would help us to develop safe reintegration strategies.

### Limitations

While this research brings new insights to research into sexual offending in sport, it also has several limitations that should be considered. This case study is based on a select sample of Belgian, adult, and male PCSO who are or were in treatment in two outpatient forensic treatment facilities in Belgium. This selection creates bias in several ways: PCSO who are convicted might be different from PCSO who are not prosecuted or convicted; the recidivism risk of people in forensic outpatient treatment facilities should in principle be lower compared to people in inpatient treatment or prisons; and it is possible this sample had received previous treatment in detention or in inpatient facilities. The generalizability of the characteristics of this small and heterogeneous sample and their concurrent risk assessment is therefore limited.

This sample only included adult, male, PCSO. While previous studies suggest that a significant portion of sexually transgressive behavior in sport is perpetrated by peer athletes, minors are not treated at the facilities where participants were recruited, and this sample therefore did not include minors. Further, no women in the treatment facilities met the inclusion criteria of this study. Little is known about the prevalence and severity of sexual offending perpetrated by women, even less so within the sport context. The Dutch-Belgian anonymous self-report study indicated that up to 12% of sexual violence against boys in sport is perpetrated by women, but these numbers are not reflected in official or clinical records (Vertommen et al., 2017). Women who have sexually offended only make up 1–2% of all people involved within the justice system for sexual offending (Cortoni et al., 2017). By necessity of the sample and the literature, this paper was also limited to adult, male PCSO in sport. However, the sport organizations and scientific field at large should acknowledge that female-perpetrated and peer-perpetrated sexually transgressive behavior constitutes a significant problem in sport and should make an effort to research this with more attention.

In terms of prevention, the static risk factors may give more insight into the mechanisms of CSA in sport compared to the dynamic risks, considering the high prevalence of multiple victims, and the often-long duration between the first offense and conviction. This might indicate that CSA in sport goes unnoticed and undetected for a long time. The dynamic risk factors are dynamic and thus changeable in treatment. This means these scores do not represent a “profile” of PCSO in sport, nor do they represent the state of the dynamic risk factors at the time of offense, but rather describe the state of that specific person under their specific, current conditions. These results cannot be used to build personality profiles or be used as a basis for selection and screening in sport staff positions. Other specific possible risk factors connected to the sport context, e.g., harsh training conditions and authoritarian coaching styles (Cense and Brackenridge, 2001), are not included in the risk assessment tools, but may be valuable in further investigation of the problem of CSA in sport.

We cannot emphasize enough that performing a forensic risk assessment is not a valid element of general screening in sport practice. It may lead to a false feeling of safety. Recidivism risk assessment is only valid to measure recidivism, i.e., re-offending, and does not measure someone's propensity to commit a first offense. These tools are developed to use in clinical practice to align treatment needs and judicial supervision of a PCSO with the recidivism risk that was assessed. The tools are developed to assess the recidivism risk of individuals and are not applicable for all people working in sport. Knowing that the vast majority of people who sexually offend are seen for the first time by the justice system (McGrath et al., 2011), these tools do not provide insights to use in a wider population.

Last, it is important to note that the researchers who completed the risk assessment used all available case file information but were not able to perform interviews with the PCSO. In common clinical practice, the assessor uses both case file information as well as an interview with the person.

## Conclusion

There is not one distinctive profile of “the” person that commits child sexual offenses, not in general society and not in sport specifically. The PCSO in sport in this sample were not the “mean, evil, creepy monsters” with many antisocial traits, as often depicted in the public discourse. People who are working in youth sport are often social and well-functioning adults who are socially embedded, integrated, and well-respected within their sport organization. While the analyses showed some prevalent risk factors, the overall expected level of sexual recidivism in this sample was lower than often assumed. These findings reiterate the importance of general primary and secondary prevention in sport and society at large.

## Data Availability Statement

The data analyzed in this study is subject to the following licenses/restrictions: Original data is owned by the forensic treatment centers. Requests to access these datasets should be directed to Tine Vertommen, Tine.Vertommen@thomasmore.be.

## Ethics Statement

The study protocol was submitted to and cleared by the Ethics Commission of the University of Antwerp (code 16/50/550).

## Author Contributions

TV: substantial contributions to the conception and design of the review, responsible for data collection, with support of undergraduate research students, analysis and interpretation of data, manuscript writing, revising of the manuscript, and final approval of the version to be published. HV: substantial contributions to the conception and design of the review, analysis and interpretation of data, manuscript writing, revising of the manuscript, and final approval of the version to be published. FM and MD: substantial contributions to the analysis and interpretation of data, manuscript writing, revising of the manuscript, and final approval of the version to be published. All authors contributed to the article and approved the submitted version.

## Conflict of Interest

The authors declare that the research was conducted in the absence of any commercial or financial relationships that could be construed as a potential conflict of interest.

## Footnotes

^1^In this paper, we use person-first language for people who have committed sexual offenses against children because of the stigmatizing effect of labeling language (Lowe and Willis, 2020).

## References

[B1] Abrams M. Bartlett M. L. (2019). #SportToo: implications of and best practice for the #MeToo movement in sport. J. Clin. Sport Psychol. 13, 243–258. 10.1123/jcsp.2018-0065

[B2] Alexander K. Stafford A. Lewis R. (2011). The Experiences of Children Participating in Organised Sport in the UK. Edinburgh: Dunedin Academic Press.

[B3] American Psychiatric Association (2013). Diagnostic and Statistical Manual of Mental Disorders: DSM-5. 5th Edn. Arlington, VA: American Psychiatric Publishing, Inc. 947p.

[B4] Bal S. Van Oost P. De Bourdeaudhuij I. Crombez G. (2003). Avoidant coping as a mediator between self-reported sexual abuse and stress-related symptoms in adolescents. Child Abuse Negl. 27:883. 10.1016/S0145-2134(03)00137-6 12951138

[B5] Barbaree H. E. Langton C. M. Blanchard R. Cantor J. M. (2009). Aging versus stable enduring traits as explanatory constructs in sex offender recidivism: partitioning actuarial prediction into conceptually meaningful components. Crim. Justice Behav. 5, 443–465. 10.1177/0093854809332283

[B6] Barth J. Bermetz L. Heim E. Trelle S. Tonia T. (2013). The current prevalence of child sexual abuse worldwide: a systematic review and meta-analysis. Int. J. Public Health 58, 469–483. 10.1007/s00038-012-0426-1 23178922

[B7] Belgisch Strafwetboek (1867).

[B8] Bjørnseth I. Szabo A. (2018). Sexual violence against children in sports and exercise: a systematic literature review. J. Child Sex Abuse 27, 365–385. 10.1080/10538712.2018.1477222 29877758

[B9] Brackenridge C. Fasting K. (2005). The grooming process in sport: narratives of sexual harassment and abuse. Auto Biogr. 13, 33–52. 10.1191/0967550705ab016oa

[B10] Brackenridge C. H. (2001). Spoilsports: Understanding and Preventing Sexual Exploitation in Sport. London: Routledge.

[B11] Brankley A. E. Helmus L. M. Hanson R. K. (2017). STABLE-2007 Evaluator Workbook Revised 2007. Ottowa, Ontario.

[B12] Brown J. Singh J. P. (2014). Forensic risk assessment: a beginner's guide. Arch. Forensic Psychol. 1, 49–59.

[B13] Bruyninckx S. (2017). Seksueel grensoverschrijdend gedrag in de sport, wat lezen we erover? Een onderzoek naar mediaberichten over seksueel grensoverschrijdend gedrag in de sport. Antwerp, Belgium: Thomas More University.

[B14] Buysse A. Enzlin P. Lievens J. T'Sjoen G. Van Houtte M. Vermeersch H.. (2013). Sexpert: basisgegevens van de survey naar seksuele gezondheid in Vlaanderen. Gent: Academia Press.

[B15] Cense M. Brackenridge C. (2001). Temporal and developmental risk factors for sexual harassment and abuse in sport. Eur. Phys. Educ. Rev. 7, 61–79. 10.1177/1356336X010071006

[B16] Cohen L. E. Felson M. (1979). Social change and crime rate trends: a routine activity approach. Am. Sociol. Rev. 44:588. 10.2307/2094589

[B17] Cortoni F. Babchishin K. M. Rat C. (2017). The proportion of sexual offenders who are female is higher than thought: a meta-analysis. Crim. Justice Behav. 44, 145–162. 10.1177/0093854816658923

[B18] de Vries Robbé M Mann R. E. Maruna S. Thornton D. (2015). An exploration of protective factors supporting desistance from sexual offending. Sex Abuse J. Res. Treat. 27, 16–33. 10.1177/1079063214547582 25143436

[B19] Elendu I. C. Umeakuka O. A. (2011). Perpetrators of sexual harassment experiences by athletes in southern Nigerian universities. South Afr. J. Res. Sport Phys. Educ. Recreat. 33, 53–63. 10.4314/sajrs.v33i1.65486

[B20] Fasting K. Brackenridge C. (2009). Coaches, sexual harassment and education. Sport Educ. Soc. 14, 21–35. 10.1080/13573320802614950

[B21] Fasting K. Brackenridge C. H. Kjølberg G. (2013). Using court reports to enhance knowledge of sexual abuse in sport. Scand Sport Stud. Forum. 4, 49–67.

[B22] Federale politie (2018). Veiligheidsmonitor 2018. Business Unit Police Management Accounting. Available online at: http://www.moniteurdesecurite.policefederale.be/assets/pdf/2018/reports/FEDERAAL_NL.pdf (accessed October 27, 2020).

[B23] Fernandez Y. Harris A. J. R. Hanson R. K. Sparks J. (2012). STABLE-2007 coding manual: Revised 2012 (unpublished scoring manual). Ottawa, Ontario: Public Safety Canada.

[B24] Finkelhor D. (1984). Child Sexual Abuse. New theory and Research. New York:, NY The Free Press. 260p.

[B25] Goethals K. De Boeck M. Dilliën T. Huys W. Nuyts A. (2020). Handboek behandeling van seksueel afwijkend gedrag. Antwerpen: Gompels & Svacina. 470 p.

[B26] Gündüz N. Sunay H. Koz M. (2007). Incidents of sexual harassment in Turkey on elite sportswomen. Sport J. 41, 1–10.

[B27] Hanson R. K. (2002). Recidivism and age: follow-up data from 4,673 sexual offenders. J. Interpers Violence 17, 1046–1062. 10.1177/08862605-0201710-02

[B28] Hanson R. K. (2018). Long-term recidivism studies show that desistance is the norm. Crim. Justice Behav. 45, 1340–1346. 10.1177/0093854818793382

[B29] Hanson R. K. Babchishin K. M. Helmus L. Thornton D. (2013). Quantifying the relative risk of sex offenders: risk ratios for Static-99R. Sex Abuse J. Res. Treat. 25, 482–515. 10.1177/1079063212469060 23264543

[B30] Hanson R. K. Bourgon G. McGrath R. J. Kroner D. D'Amora D. A. Thomas S. S.. (2017). A Five-Level Risk and Needs System: Maximizing Assessment Results in Corrections Through the Development of a Common Language. Washington DC: Justice Center Council of State Governments. 24p.

[B31] Hanson R. K. Bussière M. T. (1998). Predicting relapse: a meta-analysis of sexual offender recidivism studies. J. Consult Clin. Psychol. 66, 348–362. 10.1037/0022-006X.66.2.348 9583338

[B32] Hanson R. K. Morton-Bourgon K. (2004). Predictors of Sexual Recidivism: An Updated Meta-Analysis. Ottawa, ON: Public Works and Government Services Canada. 48p.

[B33] Hanson R. K. Morton-Bourgon K. E. (2005). The characteristics of persistent sexual offenders: a meta-analysis of recidivism studies. J. Consult. Clin. Psychol. 73, 1154–1163. 10.1037/0022-006X.73.6.1154 16392988

[B34] Hanson R. K. Morton-Bourgon K. E. (2009). The accuracy of recidivism risk assessments for sexual offenders: a meta-analysis of 118 prediction studies. Psychol. Assess. 21, 1–21. 10.1037/a0014421 19290762

[B35] Harris A. Hanson R. K. (2004). Sex Offender Recidivism: A Simple Question. Ottowa, ON: Public Safety and Emergency Preparedness Canada.

[B36] Hartill M. J. (2009). The sexual abuse of boys in organized male sports. Men Masculinities 12, 225–249. 10.1177/1097184X07313361

[B37] Helmus L. Thornton D. Hanson R. K. Babchishin K. M. (2012). Improving the predictive accuracy of Static-99 and Static-2002 with older sex offenders: revised age weights. Sex Abuse 24, 64–101. 10.1177/1079063211409951 21844404

[B38] Helmus L. M. Hanson R. K. Thornton D. Babchishin K. Harris A. (2012). Absolute recidivism rates predicted by Static-99R and Static-2002R sex offender risk assessment tools vary across samples: a meta-analysis. Crim. Justice Behav. 39, 1148–1171. 10.1177/0093854812443648

[B39] Hogan N. R. Sribney C. L. (2019). Combining Static-99R and STABLE-2007 risk categories: an evaluation of the five-level system for risk communication. *Sex Offender Treat*. 14. Available online at: http://www.sexual-offender-treatment.org/187.html

[B40] Horn J. V. Eisenberg M. Nicholls C. M. Mulder J. Webster S. Paskell C.. (2015). Stop it now! a pilot study into the limits and benefits of a free helpline preventing child sexual abuse. J. Child Sex Abuse 24, 853–872. 10.1080/10538712.2015.1088914 26701278

[B41] Kinderrechtencommissariaat (2011). Geweld, gemeld en geteld. Aanbevelingen in de aanpak van geweld tegen kinderen en jongeren. Brussel: Kinderrechtencommissariaat. 65p.

[B42] Knack N. Winder B. Murphy L. Fedoroff P. (2019). Primary and secondary prevention of child sexual abuse. Int. Rev. Psychiatry 181–94. 10.1080/09540261.2018.1541872 30917709

[B43] Lowe G. Willis G. (2020). “Sex Offender” versus “person”: the influence of labels on willingness to volunteer with people who have sexually abused. Sex Abuse 32, 591–613. 10.1177/1079063219841904 30957654

[B44] Maltz M. (2001). Recidivism. Orlando, FL: Academic Press, 252p. Available online at: https://www.academia.edu/10061829/Recidivism (accessed October 13, 2020).

[B45] Marques J. K. Day D. M. Nelson C. West M. A. (1994). Effects of cognitive-behavioral treatment on sex offender recidivism: preliminary results of a longitudinal study. Crim. Justice Behav. 21, 28–54. 10.1177/0093854894021001004

[B46] Mathews B. Lee X. J. Norman R. E. (2016). Impact of a new mandatory reporting law on reporting and identification of child sexual abuse: a seven year time trend analysis. Child Abuse Negl. 56, 62–79. 10.1016/j.chiabu.2016.04.009 27155543

[B47] Mcalinden A.-M. (2006). ‘Setting'Em Up': personal, familial and institutional grooming in the sexual abuse of children. Soc. Leg. Stud. 15, 339–62. 10.1177/0964663906066613

[B48] McGrath R. J. Lasher M. P. Cumming G. F. (2011). A Model of Static and Dynamic Sex Offender Risk Assessment. Washington, DC: United States Department of Justice. p. 96.

[B49] Op goede grond (2014). De aanpak van seksueel geweld tegen kinderen. Den Haag, Nederland: Nationaal Rapporteur Mensenhandel en seksueel geweld tegen kinderen.

[B50] Owton H. Sparkes A. C. (2015). Sexual abuse and the grooming process in sport: learning from Bella's story. Sport Educ. Soc. 22, 1–12. 10.1080/13573322.2015.1063484

[B51] Parent S. Fortier K. (2018). Comprehensive overview of the problem of violence against athletes in sport. J. Sport Soc. Issues 42, 227–246. 10.1177/0193723518759448

[B52] Parkinson P. N. Shrimpton S. Oates R. K. Swanston H. Y. O'Toole B. I. (2004). Nonsex offences committed by child molesters: findings from a longitudinal study. Int. J. Offender Ther. Comp. Criminol. 48, 28–39. 10.1177/0306624X03257246 14969114

[B53] Perkins D. Hammond S. Coles D. Bishopp D. (1998). Review of Sex Offender Treatment Programmes. United Kingdom: Broadmoor Hopstial. 31p.

[B54] Phenix A. Fernandez Y. Harris A. J. R. Helmus M. Hanson R. K. Thornton D. (2016). Static-99R Coding Rules Revised 2016. Ottowa, Canada: Public Safety Canada. 94 p.

[B55] Phenix A. Hanson R. K. Thornton D. (2000). Coding Rules for the Static-99. Ottowa, Canada: Corrections Research: Manual and Form.

[B56] Pieters J. Italiano P. Offermans A. M. Hellemans S. (2010). Ervaringen van vrouwen en mannen met psychologisch, fysiek en seksueel geweld [Experiences of Women and Men Wit Osychological, Physical and Sexual Violence]. Brussel: Instituut voor Gelijkheid van Vrouwen en Mannen.

[B57] Quinn J. F. Forsyth C. J. Mullen-Quinn C. (2004). Societal reaction to sex offenders: a review of the origins and results of the myths surrounding their crimes and treatment amenability. Deviant Behav. 25, 215–232. 10.1080/01639620490431147

[B58] Rettenberger M. Craig L. A. (2017). Actuarial risk assessment of sexual offenders. In: *The Wiley Handbook on the Theories, Assessment, and Treatment of Sexual Offending*, eds A. R. Beech and T. Ward (Chichester, UK: Wiley Blackwell), 609–41.

[B59] Rettenberger M. Craig L. A. (2020). Risk Assessment in individuals convicted of sexual offenses. In: The Wiley Handbook of What Works with Sexual Offenders, eds J. Proulx, F. Cortoni, L. A. Craig, E. J. Letourneau (Hoboken, NJ: Wiley Blackwell), 87–101.

[B60] Rintaugu E. G. Kamau J. Amusa L. O. Toriola A. L. (2014). The forbidden acts: prevalence of sexual harassment among university female athletes. Afr. J. Phys. Health Educ. Recreat. Dance 20, 974–990.

[B61] Sanghara K. K. Wilson J. C. (2006). Stereotypes and attitudes about child sexual abusers: a comparison of experienced and inexperienced professionals in sex offender treatment. Leg. Criminol. Psychol. 11, 229–244. 10.1348/135532505X68818

[B62] Schönbucher V. Maier T. Mohler-Kuo M. Schnyder U. Landolt M. A. (2012). Disclosure of child sexual abuse by adolescents: a qualitative in-depth study. J. Interpers. Violence 27, 3486–3513. 10.1177/0886260512445380 22821848

[B63] Seto M. C. (2008). Pedophilia and Sexual Offending Against Children. Washington, DC: American Psychological Association. 303p.

[B64] Seto M. C. (2017). The motivation-facilitation model of sexual offending. Sex Abuse J. Res. Treat. 31, 3–24. 10.1177/1079063217720919 28715948

[B65] Seto M. C. (2018). Pedophilia and Sexual Offending Against Children: Theory, Assessment, and Intervention. 2nd Edn. Washington, DC: American Psychiatric Publishing, Inc. 329p.

[B66] Smallbone S. Marshall W. Wortley R. (2008). Preventing Child Sexual Abuse: Evidence, Policy and Practice. United Kingdom: Routledge. 267p.

[B67] Smid W. Koch M. van den Berg J. W. (2014). Static-99R scorehandleiding: herziene uitgave. (STATIC-99R scoring manual: revised edition 2014). Utrecht, Nederland: De Forensische Zorgspecialisten. 148p.

[B68] Sport Vlaanderen (2020). Thema Sportparticipatie: Vrouw/man verhouding in het Vlaamse sportlandschap. Available online at: https://www.sport.vlaanderen/kennisplatform/thema-sportparticipatie/db-gender-in-de-sport/ (accessed October 30, 2020).

[B69] Stoltenborgh M. van IJzendoorn M. H. Euser E. M. Bakermans-Kranenburg M. J. (2011). A global perspective on child sexual abuse: meta-analysis of prevalence around the world. Child Maltreat. 16, 79–101. 10.1177/1077559511403920 21511741

[B70] Tabachnick J. (2013). Why prevention? Why now? Int. J. Behav. Consult. Ther. 8, 55–61. 10.1037/h0100984

[B71] van den Berg J. W. Smid W. Koch M. (2007). STABLE-2007 Score Handleiding. Herziene uitgave 2012 (STABLE-2007 scoring manual. Revised edition 2012. Utrecht, Nederland: De Forensische Zorgspecialisten. 278p.

[B72] van den Berg J. W. Smid W. Kossakowski J. Beek D. Borsboom D. Janssen E.. (2020). The application of network analysis to dynamic risk factors in adult male sex offenders. Clin. Psychol. Sci. 8, 539–554. 10.1177/2167702620901720

[B73] Vertommen T. Kampen J. Schipper-van Veldhoven N. Wouters K. Uzieblo K. Van Den Eede F. (2017). Profiling perpetrators of interpersonal violence against children in sport based on a victim survey. Child Abuse Negl. 63, 172–182. 10.1016/j.chiabu.2016.11.029 27923185

[B74] Vertommen T. Laureys M. Stockman D. Verhelle H. Aga N. (2019). “Jij kan de absolute top bereiken, maar alleen als je me vertrouwt”. Plegerkenmerken van seksueel kindermisbruik in de sport vanuit slachtofferperspectief. Panopticon 40, 254–272.

[B75] Vertommen T. Schipper-van Veldhoven N. Wouters K. Kampen J. K. Brackenridge C. H. Rhind D. J. A.. (2016). Interpersonal violence against children in sport in the Netherlands and Belgium. Child Abuse Negl. 51, 223–236. 10.1016/j.chiabu.2015.10.006 26516053

[B76] Ward T. Brown M. (2004). The good lives model and conceptual issues in offender rehabilitation. Psychol. Crime Law. 10, 243–257. 10.1080/10683160410001662744

[B77] World Health Organization (1999). Report of the Consultation on Child Abuse Prevention. Geneva: World Health Organization. 56p.

[B78] Wortley R. Smallbone S. (2006). Situational Prevention of Child Sexual Abuse. Monsey, NY: Criminal Justice Press. 271p.

